# Autonomous Vision-Based Object Detection and Tracking System for Quadrotor Unmanned Aerial Vehicles

**DOI:** 10.3390/s25206403

**Published:** 2025-10-16

**Authors:** Oumaima Gharsa, Mostefa Mohamed Touba, Mohamed Boumehraz, Nacira Agram

**Affiliations:** 1 Laboratory of Identification, Command, Control and Communication (LI3CUB), Department of Electrical Engineering, University of Biskra, BP 145, Biskra 07000, Algeria; oumaima.gharsa@univ-biskra.dz (O.G.); mostefa.touba@univ-biskra.dz (M.M.T.); 2 Laboratory of Energy Systems Modeling (LMSE), Department of Electrical Engineering, University of Biskra, BP 145, Biskra 07000, Algeria; m.boumehraz@univ-biskra.dz; 3 Department of Mathematics, KTH Royal Institute of Technology, 100 44 Stockholm, Sweden

**Keywords:** unmanned aerial vehicles, moving object tracking, computer vision, state estimation, object detection

## Abstract

This paper introduces an autonomous vision-based tracking system for a quadrotor unmanned aerial vehicle (UAV) equipped with an onboard camera, designed to track a maneuvering target without external localization sensors or GPS. Accurate capture of dynamic aerial targets is essential to ensure real-time tracking and effective management. The system employs a robust and computationally efficient visual tracking method that combines HSV filter detection with a shape detection algorithm. Target states are estimated using an enhanced extended Kalman filter (EKF), providing precise state predictions. Furthermore, a closed-loop Proportional-Integral-Derivative (PID) controller, based on the estimated states, is implemented to enable the UAV to autonomously follow the moving target. Extensive simulation and experimental results validate the system’s ability to efficiently and reliably track a dynamic target, demonstrating robustness against noise, light reflections, or illumination interference, and ensure stable and rapid tracking using low-cost components.

## 1. Introduction

Unmanned Aerial Vehicles (UAVs) have been steadily growing in both industrial and academic circles, and they are finding increasing applications in military contexts. Autonomous drones offer diverse applications, spanning from room cleaning and assisting disabled individuals to automating factory operations, enhancing security, facilitating transportation, enabling planetary exploration, surveillance, traffic control, etc. [1,2,3,4]. Numerous research studies on unmanned vehicles have documented advancements in autonomous systems that operate independently of human interactions [2,5]. Due to their small size, UAVs can be deployed to navigate confined spaces, asset tracking, even in scenarios where GPS signals are weak or unavailable [6]; this presents significant challenges for autonomous navigation [7] and for visual tracking systems [8].

In recent years, numerous advancements have emerged for visual detection, tracking, and autonomous navigation, spanning both indoor and outdoor settings. These innovations utilize technologies like laser range finders (LIDARs) [9], RGB-D sensors, and stereo vision to meticulously map unknown environments in three dimensions. Classical methods in visual geometry, such as SLAM [10], known as Simultaneous Localization and Mapping, and Structure from Motion (SfM) [11], rely on data from various sensors, including Kinect [12], LIDAR, SONAR, optical flow, stereo camera [8], and monocular cameras for computation. These algorithms integrate measurements from a single sensor or a combination [13], thus continually refining and updating a precise map of the UAV’s surroundings while concurrently estimating its position. Visual tracking [11] constitutes an essential sub-area of computer vision technology has been utilized and advanced in various applications, such as autonomous driving and autonomous navigation and tracking systems for unmanned aerial vehicles [3]. The challenge lies in detecting object localization, extracting accurate information, and tracking a moving object in unknown and highly variable environments, which can be influenced by factors such as noise, light reflections, or illumination interference [14], where targets may experience rapid motion or significant occlusion and even moving out-of-view.

Currently, deep learning-based detection and tracking algorithms have demonstrated high accuracy in detection and strong tracking performance [15,16,17]. Bertinetto et al. [18] introduced an end-to-end fully convolutional Siamese network for visual tracking. Li et al. [19] proposed a Siamese Region Proposal Network–based tracker trained offline on large-scale image pairs, and [20] presents a ResNet-driven Siamese tracker. For drone footage, DB-Tracker [16] is a detection-based multi-object tracker that fuses RFS-based position modeling (Box-MeMBer) with hierarchical OSNet appearance features and a joint position–appearance cost matrix, yielding robust performance in complex, occluded scenes. On the one hand, although these models offer high accuracy, they typically require high-end hardware, making them impractical for real-time applications, given the space and weight limitations of UAVs and the limited computational capacities of airborne computers and their complex computations. Within the framework of Dynamic Autonomous Tracking and Monitoring Operations (DATMO) [21], the process of identifying mobile entities is carried out through the implementation of optical flow methods, followed by their monitoring via a Kalman filter approach. This specific system was implemented on the AscTec Pelican quadrotor, which was equipped with a Firefly imaging sensor. Nevertheless, these works did not advance object surveillance further, since they were only able to conduct a basic estimation of an object’s spatial coordinates within visual frames. Kang and Cha [22] propose an autonomous UAV that replaces GPS with ultrasonic beacons, uses a deep CNN for damage detection and geo-tag detection for inspection, demonstrating high detection performance in GPS-denied zones. Ali et al. [23] integrate a modified Faster R-CNN with an autonomous UAV to map multiple damages under GPS-denied conditions, reducing false positives via streaming/multiprocessing. Waqas et al. [24] combine fiducial marker-based localization (ArUco) with deep learning and an obstacle-avoidance method to achieve autonomy in GPS-denied settings. However, methods based on beacons or visual markers (UWB/ultrasonic anchors; ArUco fields) require external infrastructure and site preparation. In [25], the authors investigate an autonomous vision-based tracking system to monitor a maneuvering target by using a rotorcraft UAV equipped with an onboard gimbaled camera. In this paper, a Kernelized Correlation Filter (KCF) tracker is combined with a redetection method within the system. An IMM-EKF-based estimator is used to estimate the states of the target. Experimental results prove that the proposed real-time vision-based tracking system gives robust and reliable tracking performance. However, when an object exhibits rapid motion or when a jump cut transpires, this vision-based tracking system encounters significant difficulties in re-establishing its tracking capabilities. While the Kernelized Correlation Filters (KCF) methodology is underpinned by a robust theoretical framework and yields commendable outcomes in experimental settings, practical applications may still experience tracking inaccuracies and a failure to recognize tracking disruptions. In instances of total occlusion, the traditional KCF framework is rendered incapable of accurately monitoring targets [26]. Furthermore, this framework exhibits effectiveness for real-time applications on an NVIDIA TK1 onboard computing platform, employing a monocular gimbal camera. In [27], a computer vision-based target tracking method is proposed for locating UAV-mounted targets, such as pedestrians and vehicles, utilizing sparse representation theory. To effectively handle partial occlusion in UAV video footage, the method integrates a Markov Random Field (MRF)-based binary support vector with contiguous occlusion constraints. The results indicate that the proposed tracker delivers enhanced precision and higher success rates. Nevertheless, the efficacy of the system may be compromised under adverse environmental circumstances, including dim illumination or densely populated backgrounds, which could adversely influence both detection and tracking precision. Furthermore, the system is explicitly engineered for the identification of pedestrians and vehicles. In instances of occlusion, the operational limitations may necessitate considerable computational resources, thereby potentially constraining real-time performance, particularly on UAVs with limited processing capabilities. Vision-based methodologies [28,29,30] predominantly concentrate on subjects situated within a specific locale or rely on periodic re-identification, thereby reducing their efficacy in rapidly evolving contexts. Furthermore, these tracking mechanisms exhibit restricted applicability, for instance, certain systems are capable of monitoring exclusively stationary ground targets [31]. This limitation arises from inadequate precision in estimating the target’s state [32]. In [33], Sean proposed a system for detecting and tracking a static spherical object and landing on a platform using a MAV equipped with a monocular camera. The spherical object was identified using a combination of HSV filtering and the Circle Hough Transform (CHT) algorithm. Notwithstanding the elevated precision and favorable success rate observed in the experimental procedures, several limitations warrant consideration: all experiments were executed within an indoor environment, and there exists a potential for the Micro Aerial Vehicle (MAV) to collide with the stationary spherical object during the search process, given the object’s immobility. Reference [34] proposes a physics-guided learning framework that embeds physical constraints into the loss to steer a quadratic neural network toward interpretable, reliable bearing-fault diagnosis when no fault samples are available, reporting high accuracy across load conditions and improved credibility versus purely data driven models. Even so, balancing physics and data losses plus GA-based weighting introduces extra hyperparameters and training overhead. Paper [35] introduces RailFOD23 (a dataset synthesized to mitigate fault-data scarcity) and EPRepSADet, a compact detector using a re-parameterizable bottleneck and lightweight self-attention; it achieves strong mAP with very low FLOPs, outperforming several state-of-the-art (SOTA) baselines on the new dataset. Still, the synthetic-to-real domain gap remains a concern—heavy reliance on AI-generated images can limit real-world transfer unless adaptation or fine-tuning is applied. In [8], a target tracking algorithm that employs correlation filters was formulated, facilitating the proficient tracking of targets that undergo considerable scale fluctuations and rapid movements. Furthermore, a redetection algorithm grounded in support vector machine (SVM) methodology has been developed to adeptly address target occlusions and losses. The estimation of target states is accomplished through the utilization of visual data, integrating an optimized Lucas–Kanade (LK) optical flow technique with an extended Kalman filter (EKF) to enhance precision. Moreover, the algorithm was investigated for tracking the target in outdoor environments. The UAV system is incapable of effectively sensing the surrounding environment to facilitate autonomous obstacle avoidance throughout the tracking procedure.

Nevertheless, constraints persist in the research investigation on quadrotor visual tracking systems. We have identified some challenging problems for moving-target tracking by UAVs:

1. The precision of the majority of drone systems is constrained by space limitations and financial considerations, resulting in most micro-UAVs being outfitted with a singular visible camera device.
2. It is imperative to acquire a precise state estimation of the target, and the UAV must be engineered to reliably monitor the maneuvering target without any preliminary information regarding it, thereby facilitating the realization of sensitive and robust tracking applications in various scenarios.
3. Consequently, the implementation of highly efficient algorithms for the detection, tracking, and estimation of pertinent states is imperative within the UAV tracking system, ensuring real-time performance despite limited onboard computation capacities. This will subsequently enable autonomous tracking in both outdoor and indoor settings.

These reasons are why we here propose a robust algorithm for object detection, tracking, and control laws, where a UAV is capable of autonomously tracking a ball without necessitating any human intervention, relying only on onboard camera sensors. This algorithm is designed to adapt efficiently and quickly to changes within the environmental context. The key contributions of the present work are summarized as follows:

1. We present a new algorithm that effectively handles noise, shadows, light reflections, and illumination interference by separating brightness from color information in the HSV color model. The algorithm operates autonomously without any user intervention or prior knowledge about the object and quickly adapts to changes in the surrounding environment.
2. An Extended Kalman Filter (EKF)-based target state estimator is presented to estimate the states of the maneuvering target at each instant. It continuously provides an estimation regarding the target’s position and re-establish tracking when the target reappears within any region of the entire frame.
3. A flight control algorithm is introduced to ensure stable tracking. This technique, based on visual tracking results, was formulated to enable the UAV to track a rapidly moving sphere while ensuring minimal power usage and enhanced real-time operational efficacy. A comprehensive framework of UAV tracking a moving target, implemented in both a simulation and a physical drone (DJI Tello drone).

The rest of this paper is organized as follows: Section 2 presents the system framework and methodology of the vision-based system. Section 3 presents an explanation of the object detection and target tracking algorithms, together with the control strategy. In Section 4, the simulation results are presented and analyzed. The validation of the developed vision-based tracking system through various experiments is covered in Section 5. The final conclusions and plans for future work are presented in Section 6.

## 2. System Overview

**Figure 1:**
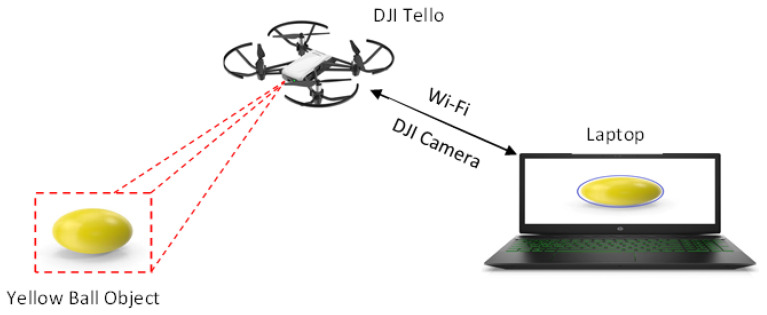
Proposed overview of the system.

The vision-based target tracking system utilizes a DJI Tello drone as the UAV platform, which communicates with a laptop via a Wi-Fi network connection. An overview of the system is shown in Figure 1.

**Figure 2:**
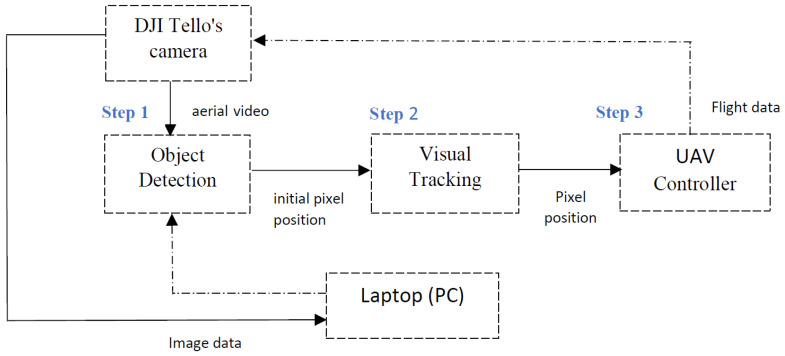
Structure of the vision and control system.

The object detection and tracking system’s work process is shown in Figure 2. The computer vision algorithm and the UAV flight controller run in real-time on a laptop.

- Step 1: The DJI Tello’s camera captures an image, which is transmitted to the laptop for detection. The position of the yellow spherical object is estimated and sent to the next module as the initial pixel position for visual tracking.
- Step 2: A switching tracking strategy is used based on the estimated states of Step 1. The visual tracking method then calculates the current pixel position of the yellow spherical object in each video frame.
- Step 3: After determining the current pixel position in Step 2, the UAV’s attitude angles and velocities for the next moment are computed by the controller module. This adjusts the UAV’s flight control parameters to ensure that it follows the yellow spherical object, keeping it within the UAV’s field of view. This process enhances the accuracy of visual tracking in Step 2.

The methods utilized in these modules will be explained in detail in the Section 3.

## 3. Methods

### 3.1. Object Detection

The object detection remains one of the most interesting and complex challenges in the Computer Vision field, a basic part of robotic perception. First of all, the ball’s position in the image plane needs to be determined via HSV segmentation defining the area in which it occurs with a 2D bounding box.

HSV color space is defined by H (hue), S (saturation), and V (value); hence, it is suited for human visual perception of colors. When required, image segmentation is more effective when using the HSV color space rather than using the RGB color space [14]. Therefore, one key benefit of the HSV color space is that its hue component enhances the algorithm’s robustness to lighting variations.

HSV represents color by a combination of three components: hue, saturation, and value [36]:

- Hue is the attribute of color, including red, blue, yellow color, etc., and its value is from 0 degrees to 360 degrees.
- Saturation is the level of color intensity, ranging from 0 to 100.
- Value is also a representation of the lightness of the color, which also falls within a range of 0–100.

The transformation of color images from RGB to the HSV color space is performed by using Equations (1)–(3) below [14].

(1) $$H=\arccos\left(\frac{\frac{1}{2}\left(2R-G-B\right)}{\sqrt{{\left(R-G\right)}^{2}-\left(R-B\right)\left(G-B\right)}}\right)$$

(2) $$S=\frac{\max(R,G,B)-\min(R,G,B)}{\max(R,G,B)}$$

(3) V=max(R,G,B)

After identifying the largest contour in the image, we then calculate the smallest enclosing circle, where we also determine its center coordinates and radius. The following equations have been used in implementing the calculation:

(4) $$C_{\mathrm{largest}}=\arg\max_{C_{i}\in \mathrm{Contours}}A\left(C_{i}\right)$$

(5) $${\left(x^{\prime }-x\right)}^{2}+{\left(y^{\prime }-y\right)}^{2}=r^{2}\;\forall \left(x^{\prime },y^{\prime }\right)\in C_{\mathrm{largest}}$$

This determines the center coordinates (x,y) and the radius r of the target ball. This initial position is required for the visual tracking algorithm.

### 3.2. Visual Tracking

#### 3.2.1. System Observations

**Figure 3:**
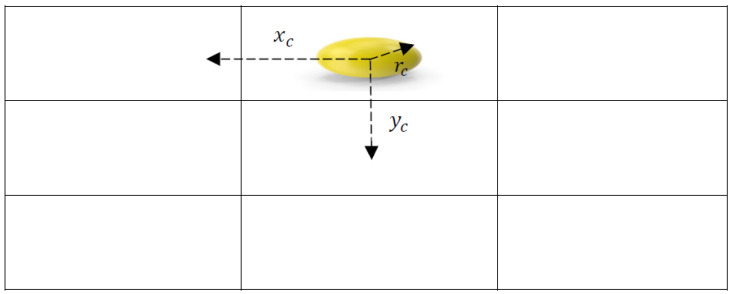
The system’s observations are represented by a vector based on the centroid position $\left(x_{c},y_{c}\right)$ and the radius $r_{c}$ of the ball in the current image frame.

The radius and centroid of the ball detected in the current image frame were calculated by the object detection algorithm [37]. These measurements are used to determine the observations or data input that will be fed into the Extended Kalman Filter. The observation vector at time *t* is defined as $O_{t}=\left[x_{c},y_{c},r_{c}\right]$, where $x_{c}$ and $y_{c}$ are the centroid coordinates of the ball and $r_{c}$ denotes the radius of the ball, see Figure 3.

#### 3.2.2. Visual Tracking by EKF

Extended Kalman Filter is an estimation technique that infers hidden states from indirect, noisy, and uncertain observations. This is done by linearizing nonlinear models around the current estimate through a first-order Taylor series expansion at every frame. Thus, it can handle noisy observations of the detection module to continuously give an estimate about the position of the template at every instant [30]. Consider the following nonlinear system, defined by the state transition and the observation model:

(6) $$x_{k}=f\left(x_{k-1},u_{k-1}\right)+w_{k-1}$$

(7) $$z_{k}=h\left(x_{k}\right)+v_{k}$$

where f(·) processes the nonlinear vector function and h(·) denotes the nonlinear observation vector function. The terms $w_{k}$ and $v_{k}$ are the process and observation noise vectors, respectively, both assumed to be zero-mean multivariate Gaussian noises. The control input vector $u_{k}$ is set to zero in this model, assuming no external acceleration input. With the functions *f* and *h* defined, the pose estimation process of the ball target can be formulated by first computing the predicted state ${\overset{˘}{x}}_{k}$ from the previous estimate, and then calculating the corrected state ${\hat{x}}_{k}$ after updating the measurement based on the predicted state.

The prediction step is as follows:

(8) $${\overset{˘}{x}}_{k}=f\left(x_{k-1}\right)$$

(9) $${\overset{˘}{P}}_{k}=F_{k}P_{k-1}F_{k}^{T}+Q_{k-1}$$

The correction step is as follows:

(10) $$S_{k}=H_{k}P_{k-1}H_{k}^{T}+R_{k}$$

(11) $$K_{k}=P_{k-1}H_{k}^{T}S_{k}^{-1}$$

(12) $${\hat{x}}_{k}=x_{k-1}+K_{k}\left(z_{k}-h\left(x_{k-1}\right)\right)$$

(13) $${\hat{P}}_{k}=\left(I-K_{k}H_{k}\right)P_{k-1}$$

In the Extended Kalman Filter (EKF), Equations (8)–(13) describe the process. Here, $Q_{k-1}$ and $R_{k}$ represent the process and measurement noise covariance matrices, respectively. $P_{k}$ is defined as the a posteriori estimate covariance matrix, $K_{k}$ denotes the Kalman gain, and $S_{k}$ is the residual covariance matrix. In these equations, the non-linear functions $f(x_{k})$ and $h(x_{k})$ are linearized, and their corresponding Jacobian matrices are denoted $F_{k}$ and $H_{k}$, respectively [38].

By adopting the ball’s position in the image plane and its global velocity as the state variables, we define the ball motion model’s state vector as follows:

(14) $$\left[\begin{array}{c} X_{c}\\ Y_{c}\\ {\dot{X}}_{c}\\ {\dot{Y}}_{c} \end{array}\right]=\left[\begin{array}{cccc} 1 & 0 & T & 0\\ 0 & 1 & 0 & T\\ 0 & 0 & 1 & 0\\ 0 & 0 & 0 & 1 \end{array}\right]\left[\begin{array}{c} X_{c-1}\\ Y_{c-1}\\ {\dot{X}}_{c-1}\\ {\dot{Y}}_{c-1} \end{array}\right]+\left[\begin{array}{c} \frac{T^{2}}{2}\\ \frac{T^{2}}{2}\\ T\\ T \end{array}\right]w_{k}$$

### 3.3. Controller

The developed vision-based control system has been designed for a quadrotor UAV equipped with a 640 × 480 pixel onboard camera, facilitating accurate real-time tracking of a moving airborne object. Maintaining the target at the center of the image plane, a PID controller is employed to regulate the UAV’s velocities and yaw angle [39]. The inputs to the PID-based controllers are the image errors e(t) at time *t*, defined as

(15) $$e_{x}={\mathrm{prd}}_{x}-{\mathrm{cen}}_{x}$$

(16) $$e_{y}={\mathrm{prd}}_{y}-{\mathrm{cen}}_{y}$$

**Figure 4:**
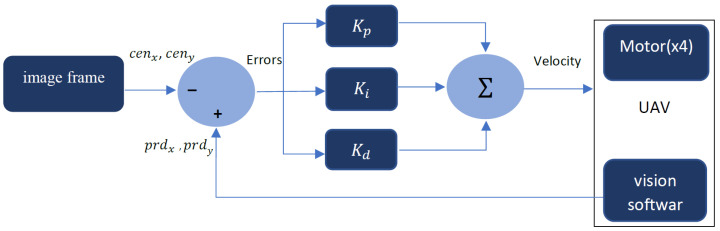
A UAV Proportional Integral-Derivative (PID) control loop system.

${\mathrm{prd}}_{x}$ and ${\mathrm{prd}}_{y}$ are the predicted state estimates of the moving object from the Extended Kalman Filter after the measurement update, where ${\mathrm{cen}}_{x}$ and ${\mathrm{cen}}_{y}$ represent the center coordinates of the current image frame, see Figure 4.

This category of controller is widely used across various applications due to its simplicity and effectiveness, with hyperparameters that are relatively easy to tune [40,41]. The controller’s output determines the motor velocity, with the parameters $K_{p}$, $K_{i}$, and $K_{d}$ adjusted for optimal performance. Consequently, the final velocity command u(t) is calculated as follows:

(17) $$u\left(t\right)=K_{p}\;e\left(t\right)+K_{i}\int_{0}^{t}e\left(\tau \right)\;d\tau +K_{d}\frac{de(t)}{dt}$$

And the corresponding transfer function is shown in Equation (18):

(18) $$u\left(s\right)=K_{p}+\frac{K_{i}}{s}+K_{d}s$$

Algorithm 1 presents the execution of the PID control pipeline for the quadrotor.
**Algorithm 1** PID Control1:prev_error←02:I_error←03:**while** true **do**4:       cur_error←predicted_state−center_of_image5:       P_error←cur_error6:       I_error←I_error+cur_error×dt7:    $\;\;\;D\text{\_}error\leftarrow \frac{cur\text{\_}error-prev\text{\_}error}{dt}$8:    $\;\;\;output\leftarrow k_{p}\times P\text{\_}error+k_{i}\times I\text{\_}error+k_{d}\times D\text{\_}error$9:       **send_command**(output)10:      prev_error←cur_error11:      **sleep**(dt)12:**end while**

Every second, the object’s positions from the current and previous video frames are extracted to calculate its speed. These calculated flight control parameters are then sent to the flight controller, enabling the UAV to follow the yellow ball accurately. To keep the ball centered in the drone’s visual frame, the drone uses the ball’s coordinates effectively. The *Y*-coordinate controls the vertical movement of the drone, allowing for it to ascend or descend to vertically align the ball within the frame. The *X*-coordinate governs the drone’s yaw, enabling it to rotate left or right around its *Z*-axis to center the ball horizontally. When the ball shifts to the left or right within the frame, the drone adjusts its yaw accordingly to maintain alignment. To approach the ball, the drone moves forward (pitch) based on the magnitude of the positional error, allowing for it to close the gap smoothly and accurately [42]. The algorithm for tracking the object is summarized in Algorithm 2, with the corresponding pseudo-code for the tracking process provided below.
**Algorithm 2** Tracking Controller1:**Inputs:** Width and Height of the onboard camera’s image, ${\mathrm{prd}}_{x}$, ${\mathrm{prd}}_{y}$2:**Initialization:**3:     ${\mathrm{cen}}_{x}\leftarrow$ Image Width / 24:     ${\mathrm{cen}}_{y}\leftarrow$ Image Height / 25:     $PID\in R^{3}$ PID parameters ($K_{p}$, $K_{i}$, and $K_{d}$)6:**Outputs:** $u\left(t\right)\in R^{3}$7:**while** true **do**8:       $e_{x}\leftarrow {\mathrm{prd}}_{x}-{\mathrm{cen}}_{x}$9:       $e_{y}\leftarrow {\mathrm{prd}}_{y}-{\mathrm{cen}}_{y}$10:      **if** $|e_{x}|\;>30$ **then**11:           **if** $e_{x}>0$ **then**12:                u(t)← left13:           **else**14:                u(t)← right15:           **end if**16:      **else if** $|e_{x}|\;\geq 7$ **then**17:           u(t)← forward18:      **end if**19:      **if** $|e_{y}|\;>30$ **then**20:           **if** $e_{y}\;>\;0$ **then**21:                u(t)← up22:           **else**23:                u(t)← down24:           **end if**25:      **else if** $|e_{y}|\;\geq 7$ **then**26:           u(t)← forward27:      **else**28:           u(t)←029:      **end if**30:**end while**

## 4. Simulation Results

**Figure 5:**
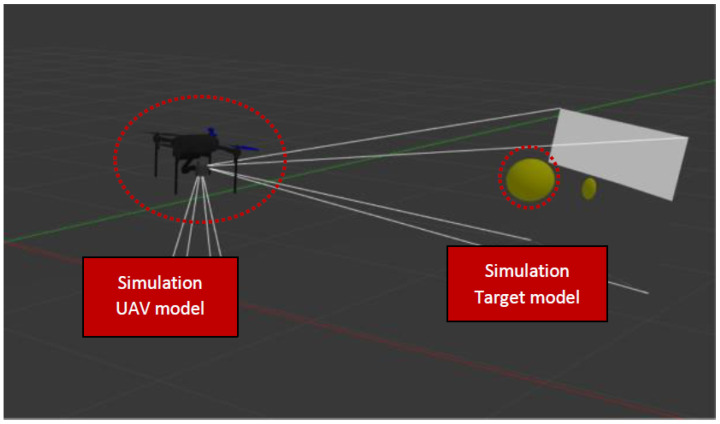
Object tracking model in a simulated environment.

This section details our tracking system, initially validated through a simulation developed using Gazebo and ROS. The tracking of the ball target within the Gazebo simulation environment is depicted in Figure 5.

This simulation has a drone with onboard cameras that are actively tracking the ball in the environment. The drone, located in the middle of the image plan, is constantly monitoring through its camera sensor to orient and fix its position relative to the ball, ensuring that it stays on track. This paper effectively demonstrates how visual perception algorithms can be used for real-time object tracking; the drone continually adjusts its flight path to maintain alignment with the ball’s movements, even when the ball follows a circular trajectory.

**Figure 6:**
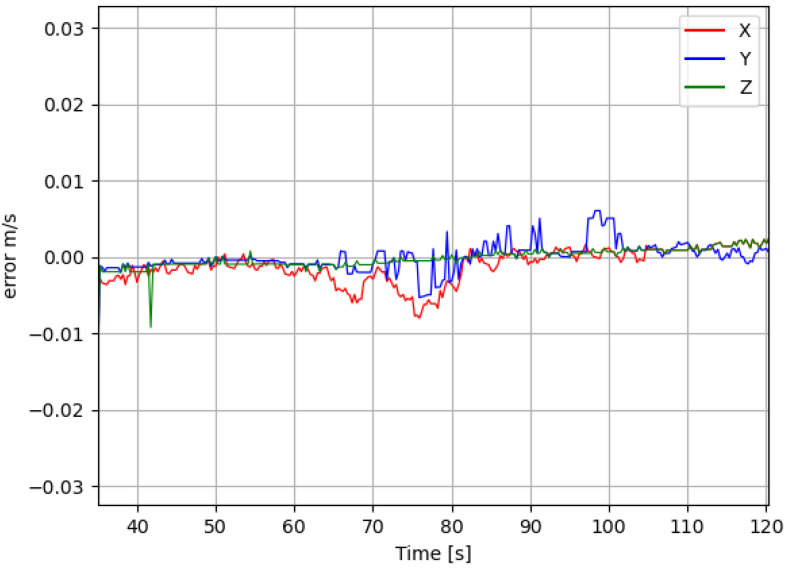
Estimated velocity error.

The estimated velocity error of the aerial target is shown in Figure 6. Between 40 and 60 s, the target remains stationary, resulting in minimal velocity error. At t=60 s, the target begins to move at a constant speed of v=1 m/s, causing a slight increase in the error. Despite this increase, the velocity error never exceeds 0.01m/s, demonstrating that the proposed state estimation algorithm remains accurate and reliable.

**Figure 7:**
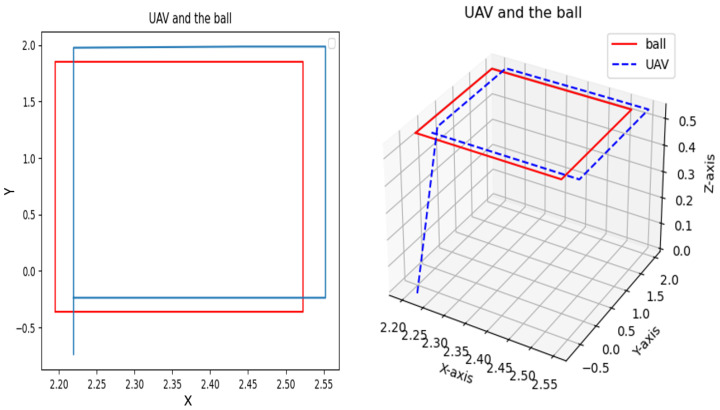
Tracking a moving target that follows a square path.

**Figure 8:**
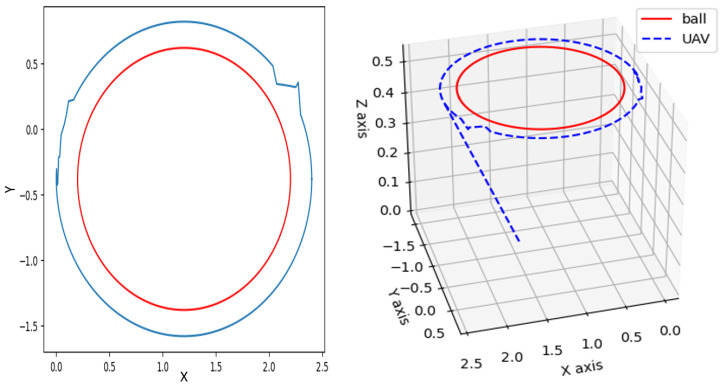
Tracking a moving target that follows a circular path.

Figure 7 and Figure 8 present the 3D trajectories of both the moving object and the quadrotor, demonstrating the UAV’s ability to successfully navigate and accurately track the target. Within a simulation environment developed using Gazebo and ROS, a ball follows two distinct trajectories a square path (Figure 7) and a circular loop (Figure 8)—while the drone autonomously tracks its motion. Leveraging advanced onboard perception algorithms, the UAV accurately detects the ball’s position and dynamically adjusts its flight path to maintain precise tracking. During the square trajectory (Figure 7), the UAV continuously updates its state estimation to ensure seamless and accurate tracking throughout the entire path. As the ball transitions to the circular trajectory (Figure 8), the drone further refines its control commands to minimize tracking errors. This simulation effectively demonstrates the UAV’s adaptability to different movement patterns and highlights the robustness of integrating vision-based algorithms within the Gazebo–ROS framework for real-time object tracking.

## 5. Experimental Results

In this section, we present results from real-world experiments using a DJI Tello Drone performing an automated tracking sequence. Our ground station utilizes an HP Pavilion Gaming 15 computer, which connects to the DJI Tello drone via a Wi-Fi network with a maximum effective range of 100 meters. In our system, the computer receives aerial video footage for further processing using a vision-based algorithm and sends commands to the DJI Tello by interfacing with its flight controller. The DJI Tello is equipped with an RGB camera sensor. During our experiment, the video was captured at a frame rate of 30 frames per second (fps), each frame having a resolution of 640 × 480 pixels.

**Figure 9:**
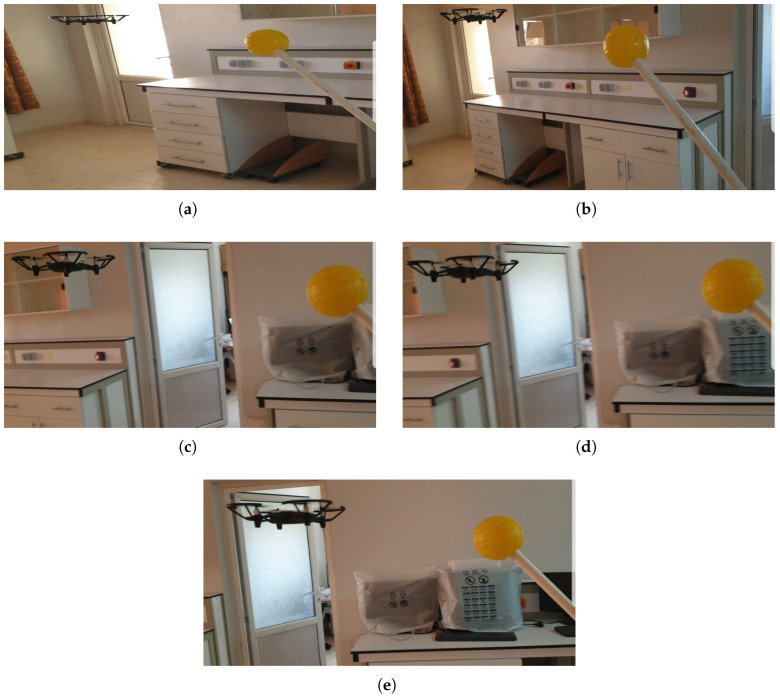
UAV tracking a moving target along a straight-line trajectory. (**a**) The UAV takes off from the ground, (**b**) approaches the ball, and (**c**) follows the ball’s motion; (**d**) continuous tracking as the ball moves; (**e**) completes the mission with successful and stable tracking of the ball.

Initially, a yellow ball was chosen as the aerial target. The UAV took off to an altitude of 1 m, positioning itself accordingly, and then detected the yellow ball in the environment while maintaining an expected separation of 5 m. The yellow ball was subsequently moved along a straight-line trajectory. In this setup, the UAV effectively detected and accurately tracked the moving target, showcasing the robust performance of our tracking system. Figure 9 depicts the UAV following the yellow ball as it travels at a constant speed along the straight line.

**Figure 10:**
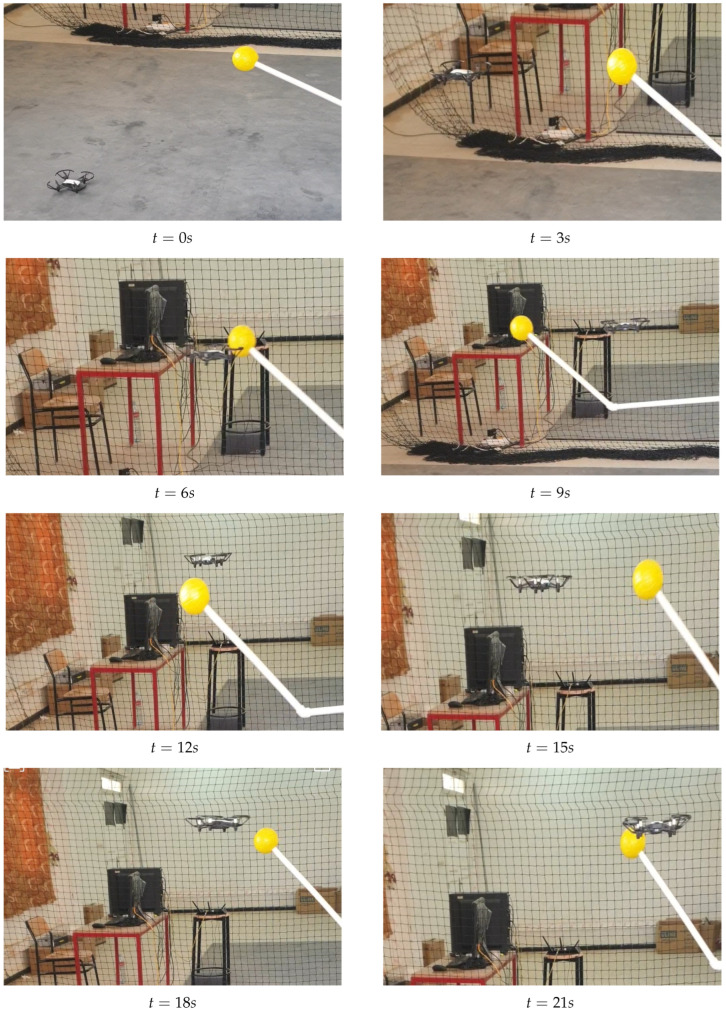
UAV tracking a moving target along the circular path.

In Figure 10, we demonstrate the detection and tracking performance by moving the yellow ball along a circular trajectory. The DJI Tello drone, programmed to follow and lock onto the yellow ball, provides a controlled scenario to evaluate our algorithms. The results showcase not only the effectiveness and robustness of our detection and tracking system, but also the reliability of our target state estimation methods under varying illumination conditions. This experimental setup validates the stability of our approach and its applicability in dynamic environments.

**Figure 11:**
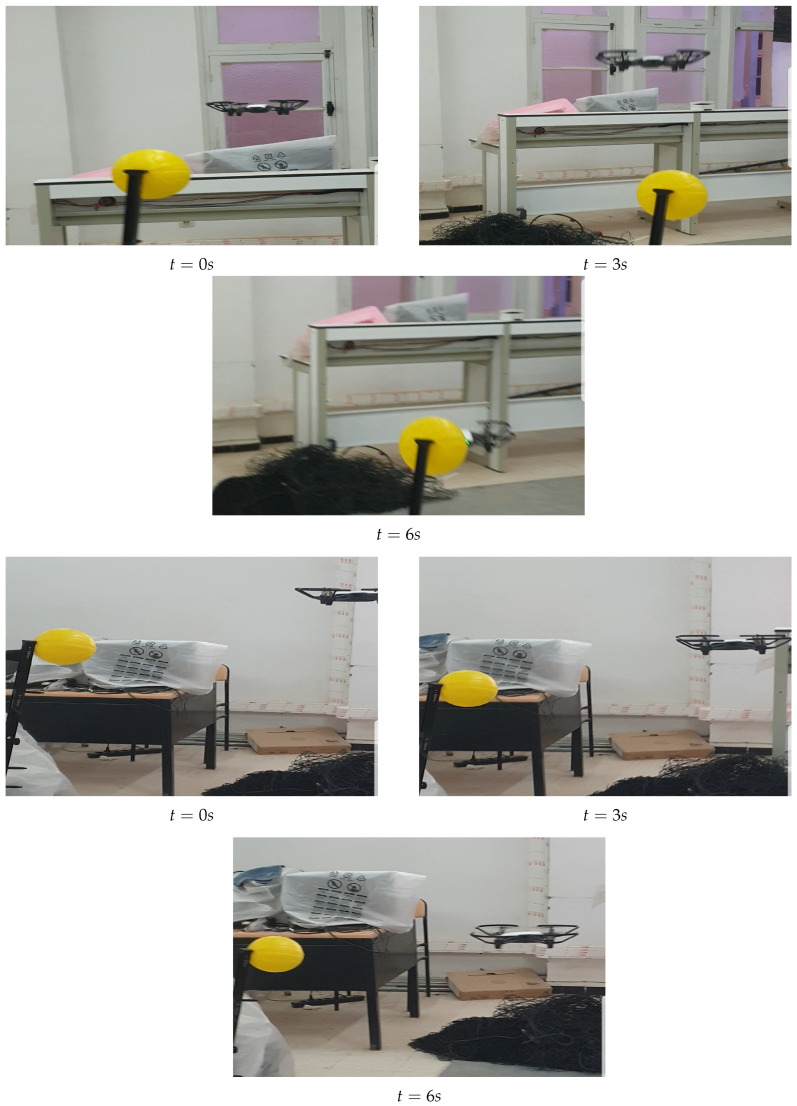
UAV tracking a moving target along random trajectories.

Due to the abundance of objects in the environment, our method remains robust and effective even when environmental conditions change, consistently ensuring accurate tracking of the yellow ball. Figure 11 illustrates the UAV and the target following random trajectories, with the ball varying its speed throughout the sequence. In this scenario, the DJI Tello drone successfully tracks the target, although a slight delay is observed during periods of rapid ball movement.

Overall, the system achieved a 90% success rate in tracking the yellow ball. In the remaining 10% of trials, failures occurred due to the ball moving outside the DJI Tello drone’s camera field of view. This study focuses on guiding an autonomous drone toward a yellow ball using visual data alone, effectively addressing the challenges posed by GPS inaccuracies and GPS-denied environments. Experimental results demonstrate that the proposed detection algorithm reliably identifies the yellow ball under varying lighting conditions and quickly adapts to changes in the surrounding environment. A total of ten flight tests were conducted to evaluate the performance and robustness of the visual tracking system.

## 6. Conclusions

In this paper, we presented a comprehensive framework for autonomous vision-based target tracking in UAVs. The proposed system utilizes an onboard RGB camera to detect and track a target using a HSV-based color segmentation approach. An Extended Kalman Filter (EKF) was employed to estimate the target’s state, ensuring continuous tracking even in the presence of temporary occlusions. Additionally, a PID controller was implemented to regulate the UAV’s motion based on visual feedback, enabling stable and efficient tracking.

Simulation and real-world experiments demonstrated the system’s effectiveness, achieving a 90% success rate in tracking the target. However, certain limitations remain. The current detection algorithm is designed specifically for yellow-colored objects, which restricts its applicability to a broader range of targets. Future improvements will focus on developing a more generalized detection algorithm capable of identifying objects of varying colors.

Another limitation is the lack of obstacle avoidance mechanisms in the current system. The UAV operates in an environment without barriers, which does not reflect real-world scenarios where obstacles may obstruct its path. Future work will integrate obstacle detection and avoidance capabilities to enhance the UAV’s autonomous navigation and tracking performance.

The proposed framework can be applied in various fields, including autonomous fruit harvesting, defense systems, and robotic applications that require precise object tracking.

Future developments will also explore optimizing computational efficiency to enable real-time performance on UAVs with limited processing power. Additionally, improving robustness to environmental variations, such as dynamic lighting conditions, will further enhance the system’s reliability in diverse scenarios. 

## Data Availability

The original contributions presented in this study are included in the article. Further inquiries can be directed to the corresponding author.
